# Leaders’ Behaviors Matter: The Role of Delegation in Promoting Employees’ Feedback-Seeking Behavior

**DOI:** 10.3389/fpsyg.2017.00920

**Published:** 2017-06-07

**Authors:** Xiyang Zhang, Jing Qian, Bin Wang, Zhuyun Jin, Jiachen Wang, Yu Wang

**Affiliations:** ^1^Beijing Key Laboratory of Applied Experimental Psychology, School of Psychology, Beijing Normal UniversityBeijing, China; ^2^Department of Human Resource Management, Business School, Beijing Normal UniversityBeijing, China; ^3^Department of Political Science, University of Rochester, RochesterNY, United States

**Keywords:** delegation, psychological empowerment, feedback seeking, power distance, positive behavior

## Abstract

Feedback helps employees to evaluate and improve their performance, but there have been relatively few empirical investigations into how leaders can encourage employees to seek feedback. To fill this gap we examined the relationship among delegation, psychological empowerment, and feedback-seeking behavior. We hypothesized that delegation promotes feedback-seeking behavior by psychologically empowering subordinates. In addition, power distance moderates the relationship between delegation and feedback-seeking behavior. Analysis of data from a sample of 248 full-time employees of a hotel group in northern China indicated that delegation predicts subordinates’ feedback seeking for individuals with moderate and high power distance orientation, but not for those with low power distance orientation. The mediation hypothesis was also supported.

## Introduction

According to a survey from SHL, a US psychometric testing company, managers spend around 14% of their time redoing tasks and correcting employees’ mistakes and this proportion is even higher in high power distance cultures such as Hong Kong (24%) and India (20%) (The Future Foundation, 2004). Clearly, people need information about their performance, but most employees do not receive timely feedback on their work. Nowadays more and more people actively seek feedback instead of waiting passively to receive it. Leaders are also aware of the situation and are finding ways to encourage employees to seek feedback.

The seeking of feedback occurs when individuals make a conscious effort to seek information about the correctness and adequacy of their behaviors and performance from others (Ashford and Cummings, 1983). Supervisors’ feedback can help employees to evaluate and improve their performance as well as clarifying role expectations (Renn and Fedor, 2001; Ashford et al., 2003; Whitaker et al., 2007). Since Ashford and Cummings (1983) proposed the feedback-seeking construct there have been numerous studies exploring its antecedents. Some studies emphasize the role of individual characteristics in tendency to seek feedback, for example there is evidence that desire for useful information, motivation to manage impression, learning goal orientation and high self-esteem drive employees to seek feedback at work (e.g., Fedor et al., 2001; Tuckey et al., 2002; Bernichon et al., 2003; Anseel et al., 2015).

Recently researchers have started to explore the contextual antecedents of feedback-seeking, in particular how leaders could influence employees’ seeking of feedback. For example, in a study with 132 participants Levy et al. (2002) found that the presence of a transformational leader was positively associated with intention to seek feedback. They suggested that exposure to a certain leader and the perception that a leader has certain characteristics are important determinants of tendency to seek feedback from supervisors. Qian et al. (in press) proposed that authentic leadership promotes the seeking of feedback by influencing employees’ beliefs about the value of feedback and cost of seeking it. Authentic leadership also promotes positive emotions amongst employees and thus encourages them to seek feedback from supervisors.

In this research we focused on delegation, a managerial technique whose potential to change the work context is already recognized. As more and more companies adopt flat or non-hierarchical organizational structures, delegation will become an increasingly popular managerial technique (Kastelle, 2013). Many successful companies, including Google and Facebook, attract talent with a more relaxed management culture in which authority is delegated and subordinates are more involved in decision-making (Garvin, 2013). When power and authority are delegated to employees they have more freedom to work autonomously and experience a range of positive outcomes such as higher job satisfaction, organizational commitment, innovative behavior and task performance (Chen G. et al., 2007). Delegation also motivates subordinates to enhance their skills and expertise (Uhl-Bien et al., 2000). As yet, however, there is a lack of empirical evidence that delegation is associated with the seeking of feedback. In this study, therefore, we tested the hypothesis that delegation would increase employees’ feedback seeking from supervisors.

We also set out to examine the mechanism through which delegation influences followers’ feedback-seeking behavior. Psychological empowerment has been defined as increased intrinsic task motivation that is manifested in cognitions that reflect an individual’s active orientation to his or her work role (Spreitzer, 1995, p. 1443). Numerous empirical studies have shown that psychological empowerment is the mechanism underlying leaders’ influence on outcome variables (e.g., Avolio et al., 2004; Alge et al., 2006; Chen G. et al., 2007; Dewettinck and van Ameijde, 2011). More importantly, it has been shown that psychological empowerment plays an important role in the feedback-seeking process (e.g., Chen Z. et al., 2007; Huang, 2012). In this study, we have responded to this call by examining psychological empowerment as a potential mediator of the relation between delegation and feedback seeking. We hypothesized that psychological empowerment of subordinates would predict feedback-seeking behavior and would mediate the relationship between delegation and seeking of feedback from supervisors.

Additionally, we aim to identify the limits to the influence of delegation on feedback-seeking behavior by examining whether this relationship was moderated by an individual-level variable, namely power distance. Power distance is defined as the extent to which one accepts the unequal distribution of power in society and organizations (Hofstede, 1980) and has been explored as an individual-level construct and at cultural level (e.g., Kirkman et al., 2009). It is considered to have an important influence on reactions to leaders (Kirkman et al., 2009). It is not surprising, therefore, that many studies have attempted to identify how power distance moderates the influence of leadership behaviors and managerial techniques (e.g., Farh et al., 2007; Liu and Liao, 2013; Qian and Li, 2016). In this study we examined how power distance moderates the impact of delegation on feedback-seeking behavior.

As Bass said, “delegation implies that one has been empowered by one’s superior to take responsibility for certain activities” (Bass, 1990, p. 437). Delegation is closely related to empowerment. Empowerment is a motivational concept related to self-efficacy. People experience psychological empowerment when they feel responsible for meaningful tasks. They also feel empowered when they believe they are competent and make a difference. In earlier works empowerment was conceptualized as a leader behavior that was similar to delegation (e.g., Locke and Schweiger, 1979; Miller and Monge, 1986; Cotton, 1988, 1993), but more recently it has been defined as a constellation of psychological states experienced by employees (e.g., Sigler and Pearson, 2000; Niehoff et al., 2001; Randolph and Kemery, 2011; Frazier and Fainshmidt, 2012; Maynard et al., 2014). Previous work has demonstrated that leadership empowering behavior and managerial empowerment practice is positively related to psychological empowerment (Dewettinck and van Ameijde, 2011; Randolph and Kemery, 2011). We argue that delegation is an antecedent of psychological empowerment.

When responsibility or authority is delegated to employees they usually find that they are faced with a challenging, complex task to tackle independently; the task may require a high level of skill and may have significance. Thus delegation may make subordinates feel that their job is meaningful and they are responsible for work outcomes. Managers are more likely to delegate to subordinates who have worked for them for a relatively long time and are particularly competent; they are also more willing to delegate to subordinates who are also managers (Yukl and Fu, 1999). Therefore, when subordinates are delegated, they may feel trusted, organisationally important, and higher status within organization (Gardner et al., 2004; Chen and Aryee, 2007). Delegation may also boost subordinates’ self-esteem and make them believe that they are capable of performing tasks successfully and that their behavior makes a difference. Delegation enables subordinates to exercise self-direction and control, provides employees with meaning, perceptions of self-efficacy and self-determination and the perception that they make an impact, all of which have been identified as key ingredients of empowerment (Thomas and Velthouse, 1990; Spreitzer, 1995).

*Hypothesis 1: Delegation is positively related to psychological empowerment.*

Psychological empowerment is essential to organizational effectiveness (Mathieu et al., 2006). Empowered employees are more innovative, happier, and more productive. Motivation, loyalty, problem-solving performance and coordination between functions also improve with psychological empowerment (e.g., Spreitzer, 1995; Sigler and Pearson, 2000; Niehoff et al., 2001; Laschinger et al., 2002; Molix and Bettencourt, 2010; Zhang and Bartol, 2010; Seibert et al., 2011). However, there is little empirical evidence that psychological empowerment increases the tendency to seek feedback from supervisors. We argue that empowerment should increase the seeking of feedback from supervisors in several ways.

First, empowered employees are motivated and actively oriented to their work role. They feel that their behavior makes a difference and they have responsibility for tasks (Spreitzer, 1995). They may, therefore, actively search for ways to improve the quality of their work. From an instrumental perspective, feedback is a valuable way of acquiring useful information (Ashford et al., 2003). Herold and Greller (1977) found that workers viewed feedback as an important way of improving the quality of their work because it gave them information about how well others thought they were doing their job. Subordinates may choose to seek feedback more frequently as a way of improving the quality of their work (VandeWalle et al., 2000; Ashford et al., 2003). Accordingly, empowered employees may actively seek feedback as a way of improving their performance.

Second, empowerment can be an indication of a relatively strong relationship between employer and subordinates. Specifically, empowerment may indicate that there is mutual trust and frequent interaction between employer and subordinates. Numerous empirical studies have found that employee empowerment is positively associated with trust in supervisors (Laschinger and Finegan, 2005; Moye and Henkin, 2006; Ergeneli et al., 2007; Huang, 2012). Zhu et al. (2004) proposed that empowered employees are more likely to trust their leaders and feel commitment to the organization. It has been suggested that employees who feel empowered usually trust their employers, which usually enhances communication and improves problem-solving processes (Willemyns et al., 2003). Trust is a determinant of the feedback-seeking behavior of subordinates (Barner-Rasmussen, 2003). Mutual trust and respect from leaders for subordinates’ ideas can increase subordinates’ feedback-seeking by lowering the perceived cost of feedback (VandeWalle et al., 2000). Empowered employees are also more confident and feel that they are competent and can make an impact. Chen G. et al. (2007) proposed that employees who feel empowered contribute more actively when involved in groups of higher organizational status. It may also enhance their relationship with supervisors which were shown to be crucial to subordinates’ seeking of feedback (Barner-Rasmussen, 2003). Having a supportive leader was shown to increase the frequency with which employees sought feedback (Williams et al., 1999).

Finally, empowered employees are less afraid of receiving negative feedback. Scholars assume that performance feedback is a complex, multidimensional construct that encompasses both positive and negative feedback (Van Dijk and Kluger, 2011). People who receive negative feedback may deny or distort it. Because it can be perceived as a threat and may cause the recipient to lose face, or damage his or her self-esteem (Bernichon et al., 2003). It is inevitable that sometimes the quality of one’s work will not meet the supervisor’s expectations and will elicit negative feedback. In this situation employees may be required to make lots of revisions and face considerable criticism, with the result that their self-esteem suffers. Empowered employees feel competent, feel that they make an impact and usually have high self-esteem. They tend to seek feedback to achieve goals even if that feedback is negative. In contrast, people with low self-esteem are concerned with self-protection and less able to tolerate negative feedback (Bernichon et al., 2003). Empowered employees tend to be confident about their work and hence are less worried about the prospect of negative feedback and are thus more likely to seek feedback from supervisors.

*Hypothesis 2: Psychological empowerment is positively related to the seeking of feedback from supervisors.*

Delegation can provide nourishing conditions necessary for feedback-seeking by psychologically empowering subordinates and motivating them to improve their work quality. When delegated authority and responsibility, subordinates may feel they are trusted and organizationally important (Gardner et al., 2004; Pierce and Gardner, 2004; Chen and Aryee, 2007). It will also boost their self-esteem and make them believe their supervisors consider them to be able, task-competent, and need-satisfying (Blau and Alba, 1982; Sashkin, 1984). Therefore, they are motivated to enhance their work quality. As a consequence, subordinates may actively seek feedback, which is useful for employees to evaluate their work and improve their performance (Ashford et al., 2003).

If employees do not feel empowered, delegation itself may not lead to feedback-seeking behavior. When delegated tasks or authority, employees will experience more autonomy and task identity, which makes them feel more responsible for results and more sensitive to negative feedback (Krasman, 2013). Therefore, feedback-seeking behavior may not increase. However, if delegation empowers employees psychologically, employees may seek feedback more. This is because employees will feel greatly motivated to achieve the task with high quality. Delegation can boost their self-esteem and employees will be less sensitive to negative feedback with more confidence to seek feedback proactively.

Moreover, previous research has highlighted the importance of the psychological process and motivation underlying the feedback-seeking process (e.g., Ashford and Cummings, 1983; Morrison and Bies, 1991; Tuckey et al., 2002). Empirical studies have suggested that psychological empowerment plays an important role in the feedback-seeking process (e.g., Chen G. et al., 2007; Huang, 2012). For example, Huang (2012) suggested psychological empowerment is positively related to feedback-seeking behavior. Anseel and Lievens (2007) called for more research on integrating the motives traditionally used in the literature on seeking feedback (i.e., ego-based or image-based motivation) with the social psychological literature on self-motivation. We have responded to this call by examining psychological empowerment as a potential mediator of the relation between delegation and feedback-seeking. Accordingly,

*Hypothesis 3: Psychological empowerment mediates the relationship between delegation and seeking feedback from supervisors.*

We also included power distance in our study in order to explore whether it moderated the relationship between delegation and feedback-seeking behavior. There is considerable individual-level variation in power distance within organizations (Clugston et al., 2000; Farh et al., 2007). Earlier research suggested that power distance beliefs affect how people react to particular leadership styles or behaviors (Eylon and Au, 1999; Brockner et al., 2001; Kirkman et al., 2009). Following previous studies, we operationalised power distance at the individual level as whether or not the employee accepted the unequal distribution of power in the organization (Clugston et al., 2000, p. 9). Employees with high power distance regard the uneven social distribution of power as natural and even desirable. They accept status differences and have formal, less personal relationships with their supervisors (Tyler et al., 2000). People with low power distance believe that equality and justice are important and that power should be more evenly distributed (Mueller and Wynn, 2000; Yang et al., 2007).

We hypothesized that power distance moderates the relationship between delegation and seeking of feedback, and specifically that this relationship will be stronger in the context of high power distance. High power distance employees believe that people in powerful positions have more decision-making power and they are more likely to be willing to comply with the decisions of powerful others (Farh et al., 2007; Chen and Aryee, 2007). They may value leaders’ delegation of power more than low power distance individuals and delegation may motivate them to perform better and improve their skills and expertise (Uhl-Bien et al., 2000). Hence delegation may prompt high power distance employees to seek feedback more frequently, in order to use the information to improve their skills and performance.

*Hypothesis 4: Power distance moderates the relationship between delegation and seeking of feedback such that delegation has more impact on feedback-seeking in high power distance individuals than in lower power distance individuals.*

## Materials and Methods

### Participants and Procedure

The participants were employees of a hotel group in northern China. Different questionnaires were administered to supervisors and subordinates to minimize common method bias. With the help of the hotel’s human resources department, we distributed questionnaires to 64 supervisors at a training held for supervisors. Then, assisted by the human resources department, we randomly selected five subordinates for each supervisor, totalling 320 subordinates. We assigned an identification number to each questionnaire in order to match subordinates with evaluations of their immediate supervisors. To ensure confidentiality, we asked all respondents to seal their completed questionnaire in an envelope and return it to the research team at an all-employee company meeting held 2 weeks after the questionnaires were distributed. The day before the meeting, we sent text messages to participants instructing them to place the envelope containing their completed questionnaire in a special box at the entrance of the meeting venue. Subordinates were asked to provide information about their demographic variables, perceived supervisors’ delegation, psychological empowerment and power distance, while their supervisors were asked to rate the subordinates’ feedback seeking behavior.

Of the questionnaires distributed to 64 supervisors, 57 questionnaires were completed, representing response rates of 89.06%. For those subordinates who have evaluation from their supervisors, 248 questionnaires were completed, representing response rates of 77.5%. For subordinates, the majority of the participants were men (62.1%), the mean age of respondents was 32.58 years (*SD* = 8.28), and their mean organizational tenure was 6.31 years (*SD* = 3.99). The majority of subordinate respondents (75.8%) had a bachelor’s degree, 8.5% of had only a high school diploma and the rest had a master’s degree or higher degree.

### Measures

The original versions of the instruments we used were written in English. We used a standard translation and back-translation procedure (Brislin, 1980) to produce equivalent Chinese versions. The Chinese questionnaire was subsequently pilot-tested on 20 employees of the participating organization who were excluded from the final sample.

**Delegation** was assessed using a six-item scale developed and validated by Schriesheim and Neider (1988) with response options ranging from 1 (none of the time) to 5 (always). Sample items include “My supervisor does not require that I get his/her input or approval before making decisions” and “My supervisor permits me to get the necessary information from him/her and then make my own decisions.” The scale’s reliability was 0.84.

**Psychological empowerment** was assessed using Spreitzer Spreitzer’s, (1995, 1996)’s Psychological Empowerment Scale. Responses were given using a five-point Likert scale ranging from 1 (strongly disagree) to 5 (strongly agree). Sample items include “The work I do is meaningful” and “I am confident in my ability to do my job.” High scores indicate greater perceived psychological empowerment. The overall reliability of the scale was 0.72.

**Feedback-seeking behavior** was assessed using a five-item scale validated by VandeWalle et al. (2000). We asked supervisors how frequently their subordinates asked for feedback about (1) overall work performance, (2) technical performance in the job, (3) the supervisors’ role expectations of them, (4) social behavior and (5) whether supervisors felt that the subordinate’s values and attitudes were appropriate for the firm. Responses were given using a five-point Likert scale ranging from 1 (never) to 5 (always). The scale’s reliability was 0.83.

**Power distance** was assessed using a six-item measure of power distance developed by Dorfman and Howell (1988). Responses were given using a five-point Likert scale ranging from 1 (strongly disagree) to 5 (strongly agree). Sample items are “It is frequently necessary for a manager to use authority and power when dealing with subordinates,” “Managers should avoid off-the-job social contacts with employees” and “Employees should not disagree with management decisions.” The scale’s reliability was 0.82.

### Data Analysis Strategy

First we used AMOS 21.0 to examine the extent to which our data were affected by common method bias. Next we examined descriptive statistics, correlations among study variables and demographic group differences in the study variables. Then we used hierarchical regression to control for variance in the demographic variables and test hypotheses 1–4. Structural equation modeling was used to confirm the results of the mediation test. All study variables were centered before indirect effects tests.

## Results

### Confirmatory Factor Analysis

We used AMOS to perform confirmatory factor analysis in order to determine the proportion of variance attributable to use of a common method. Following the recommendations of Podsakoff et al. (2003) and the method used by Elangovan and Xie (1999), we controlled for the effects of an unmeasured latent methods factor. The AMOS analyses were run on 28 indicators with 4 trait factors and a methods factor. To demonstrate that the results are not due to method effects, the addition of a method factor to a t-traits factor model must not significantly improve the fit of the model. The overall chi-square fit statistics for the t-traits factor model were: χ^2^(344) = 2060.60, *p* = 0.00; GFI = 0.703. The overall chi-square fit statistics for the model containing the t-traits factors plus the methods factor were: χ^2^(343) = 1721.54, *p* = 0.00, GFI = 0.698. Although the overall chi-squared statistic was significant for both models, the incremental fit index yielded a rho of 0.032 which suggests that adding the methods factor did not improve model fit significantly (Bentler and Bonett, 1980). In summary, this suggests that common method bias was not a serious problem in our research. Respondents did differentiate between the variables and that the results obtained in the analyses are reliable.

### Descriptive Statistics

**Table 1** presents means, standard deviations and correlations between the study variables. We also examined associations between employees’ gender, age, education level and corporate tenure and all the variables of interest. Delegation was positively associated with psychological empowerment (*r* = 0.26, *p* < 0.01) and with seeking of feedback (*r* = 0.31, *p* < 0.01). Psychological empowerment was also positively associated with seeking of feedback (*r* = 0.36, *p* < 0.01). Seeking feedback was positively associated with education (*r* = 0.14, *p* < 0.05) and power distance was negatively associated with education (*r* = -0.19, *p* < 0.05). The results of the correlation analysis generally supported hypotheses 1–3.

**Table 1:** Means, standard deviations, and correlations.

	*M*	*SD*	1	2	3	4	5	6	7	8
(1) Gender	1.38	0.49								
(2) age	32.58	8.28	0.00							
(3) Education	2.08	0.50	000	0.09						
(4) Company tenure	6.30	3.98	-0.01	0.48^∗∗^	0.07					
(5) Delegation	4.21	0.49	-0.06	0.04	0.08	-0.03	(0.83)			
(6) Psychological empowerment	3.88	0.34	-0.09	0.06	0.12	0	0.26^∗∗^	(0.84)		
(7) Power distance	3.13	0.87	0.06	-0.03	-0.19^∗∗^	-0.08	-0.12	-0.07	(0.72)	
(8) Feedback-seeking behavior	3.82	0.61	-0.01	0.12	0.14^∗^	0.1	0.31^∗∗^	0.36^∗∗^	0.02	(0.82)

**n* = 248; ^∗^*p* < 0.05, ^∗∗^*p* < 0.01; Education: 1 = high school degree or an associated degree, 2 = bachelor degree, 3 = master degree, 4 = doctoral degree; Gender: 1 = male, 2 = female*.

### Hypothesis Testing

Further analyses were conducted to provide a better estimate of how well delegation predicted the outcome variables and the extent to which these relationships were mediated by psychological empowerment. We used Preacher and Hayes’s (2008) procedure to examine whether psychological empowerment mediated the association between delegation and feedback-seeking behavior. They propose three criteria that must be met before mediation can be inferred. First, the independent variable should be correlated with mediator variable. Second, after controlling for the effect of the independent variable on the dependent variable, there should be a correlation between the mediator variable and dependent variable. Finally, the independent variable should have an indirect effect on the dependent variable. Before the analyses all continuous predictors were centered (Aiken and West, 1991).

As shown in **Table 2** delegation predicted the feedback-seeking behavior after controlling for the effect of demographic variables (gender, age, education and tenure) (Model 3: β = 0.31, *p* < 0.001). Similarly, delegation predicted psychological empowerment (Model 1: β = 0.24, *p* < 0.001), and psychological empowerment predicted the seeking of feedback (Model 2: β = 0.35, *p* < 0.001). Thus, hypotheses 1 and 2 were supported.

**Table 2:** Regression results for hypothesis tests.

	Psychological empowerment as dependent variable	Feedback seeking behavior as dependent variable			
	Model 1	Model 2	Model 3	Model 4	Model 5
Gender	-0.08	0.02	0.01	0.03	0.02
Age	0.05	0.05	0.06	0.04	0.05
Education	0.09	0.09	0.11	0.08	0.12
Tenure	-0.02	0.07	0.08	0.09	0.09
Delegation	0.24^∗∗∗^		0.31^∗∗∗^	0.24^∗∗∗^	0.31^∗∗∗^
Psychological Empowerment		0.35^∗∗∗^		0.29^∗∗∗^	
Power Distance					0.10
Delegation × Power Distance					0.16^∗^
*R*^2^	0.08	0.15	0.13	0.20	0.16
Δ*R*^2^				0.08	0.03
*F*	4.38^∗∗^	8.64^∗∗∗^	6.95^∗∗∗^	10.23^∗∗∗^	6.36^∗∗∗^
Δ*F*				23.4^∗∗∗^	4.39^∗∗^

*n = 248; ^∗^*p* < 0.05, ^∗∗^*p* < 0.01, ^∗∗∗^*p* < 0.001; values are standardized coefficient; Δ*R*^2^ and Δ*F* showed in models 4 and 5 is based on model 3*.

Hierarchical regression was conducted to test for potential mediation of the relationship between delegation and feedback-seeking by psychological empowerment. First we entered the control variables, then delegation and finally psychological empowerment. As shown in Model 4 in **Table 2**, psychological empowerment predicted feedback-seeking (β = 0.29, *p* < 0.01), whilst delegation predicted the dependent variable (β = 0.24, *p* < 0.001) albeit less powerfully (Model 3: β = 0.31, *p* < 0.001). These findings are indicative of partial mediation; delegation had an indirect effect on feedback-seeking that was mediated by psychological empowerment as well as having a direct effect on feedback-seeking. Thus, hypothesis 3 was supported.

**FIGURE 1:**
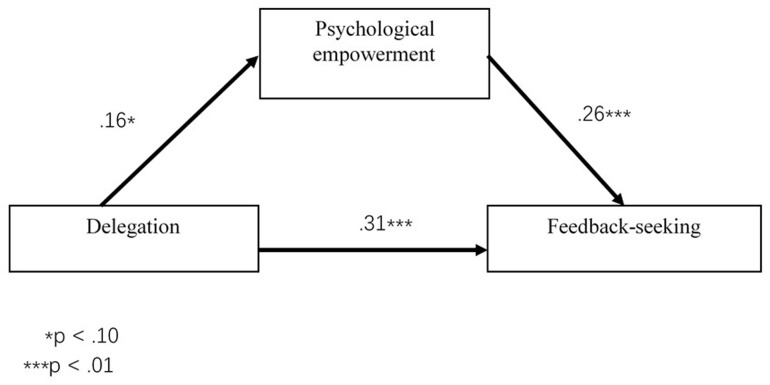
Results of structural equation modeling on the mediating effect of psychological empowerment.

We used structural equation modeling to confirm the results of the mediation analysis. The results of structural equation modeling were similar to those of the hierarchical regression analysis. We assessed the overall fit of the model to the data using chi-squared and the goodness-of-fit index (GFI), normed fit index (NFI), CFI, RMSEA and RMR. The goodness-of-fit statistics supported the conclusion that the hypothesized model was an adequate fit for the data (χ^2^ = 36.67, *p* < 0.05, df = 23; χ^2^/df = 1.594; CFI = 0.98; NFI = 0.95; GFI = 0.97; RMSEA = 0.049; RMR = 0.019). These results provide compelling evidence that psychological empowerment is a mediator of the relationship between delegation and seeking of feedback. **Figure 1** shows that the coefficient of the path from delegation to psychological empowerment was marginally significant (β = 0.16, *p* = 0.06), and the coefficients of the paths from delegation (β = 0.26, *p* < 0.01) and psychological empowerment (β = 0.31, *p* < 0.01) to feedback seeking were significant.

**FIGURE 2:**
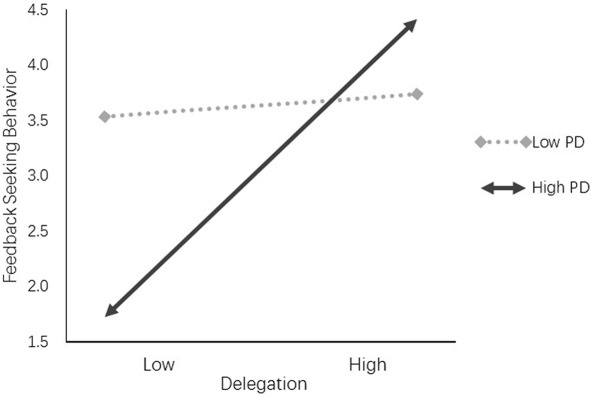
Delegation and feedback seeking by power distance.

We tested hypothesis 4 using the procedure described by (Olson et al., 2007). We created linear–linear interaction terms by multiplying the proposed moderator (power distance) by delegation. After entering the main effects and control variables into the equation, the multiplicative terms were added. The regression weights for the multiplicative terms were then examined for significance. The results are presented as model 5 in **Table 2**. Power distance moderated the influence of delegation on feedback seeking behavior (β = 0.14, *p* < 0.05). As shown in **Figure 2**, delegation is more likely to increase the seeking of feedback for individuals with medium and high power distance, than for individuals with low power distance. Thus, hypothesis 4 was supported.

## Discussion

We developed and tested a model linking delegation with employees’ seeking of feedback from supervisors by investigating the underlying mechanisms of the associations and their boundary conditions. The results revealed that: (1) delegation is positively associated with psychological empowerment; (2) psychological empowerment is positively associated with feedback-seeking; (3) psychological empowerment mediates the relationship between delegation and feedback-seeking and (4) power distance moderates the relationship between delegation and psychological empowerment.

Our research contributes to the literature on seeking feedback and extends previous research in the following ways. First, as Bass said, “delegation implies that one has been empowered by one’s superior to take responsibility for certain activities” (Bass, 1990, p. 437). It used to be thought that delegation was very closely related to the concept of empowerment (e.g., Locke and Schweiger, 1979; Miller and Monge, 1986; Cotton, 1988, 1993), but a clear distinction is now drawn between them (e.g., Sigler and Pearson, 2000; Niehoff et al., 2001; Randolph and Kemery, 2011; Frazier and Fainshmidt, 2012; Maynard et al., 2014) and there have been few studies demonstrating an association between them. Our findings provide evidence that delegating power to an individual is positively associated with the psychological empowerment of that individual.

Second, psychological empowerment is essential to organizational effectiveness (Mathieu et al., 2006): it is positively associated with innovation, happiness, production, motivation, loyalty, effective problem solving and coordination between functions (e.g., Spreitzer, 1995; Sigler and Pearson, 2000; Niehoff et al., 2001; Laschinger et al., 2002; Molix and Bettencourt, 2010; Zhang and Bartol, 2010; Seibert et al., 2011). There is, however, little empirical research demonstrating that psychological empowerment increases the frequency with which employees seek feedback from supervisors. Our findings extend previous research by demonstrating that psychological empowerment is positively associated with the seeking of feedback.

Third, our research has significant theoretical implications for the emerging strand of research into how leaders can influence employees’ feedback-seeking behavior. Earlier research examined the extent to which individual characteristics such as desire for useful information, learning goal orientation and high self-esteem drive employees to seek feedback at work (e.g., Fedor et al., 2001; Tuckey et al., 2002; Bernichon et al., 2003; Anseel et al., 2015). However, there have been relatively few empirical studies into leader behaviors and managerial techniques that encourage employees to seek feedback. As the popularity of non-hierarchical organizational structures has increased, delegation has become an increasingly popular way of empowering employees. It is becoming more and more common for successful companies to attract employees by giving them more freedom to work autonomously and greater involvement in decision-making. We investigated the extent to which a supervisor’s delegation of power increased subordinates’ tendency to seek feedback and assessed the extent to which this relationship was mediated by psychological empowerment and thus filled the gap in the literature.

Previous research suggested that power distance can provide a boundary condition for leadership’s influence on subordinates’ attitudes and behavior (e.g., Eylon and Au, 1999; Kirkman et al., 2009; Fock et al., 2012). It may influence the relationship between delegation and subordinates’ seeking of feedback, but the nature of the relationship remains unknown. Our results showed that power distance moderates the effect of delegation on seeking of feedback. Specifically, delegating power to subordinates with a moderate to high power distance increases the frequency with which they seek feedback, but delegating power to subordinates with a lower power distance does not. This result indicates that individuals with a higher power distance tend to respond more positively to delegation. Because they regard the uneven social distribution of power as natural and even desirable, when leaders delegate, they may value this action more and take it more seriously than low power distance individuals. Thus, they may seek feedback more frequently in the following process.

### Practical Contributions

Our theoretical model and empirical findings have important implications for managers and human resources practitioners. First, our model suggests that delegation of power by a supervisor can be psychologically empowering for employees and thus increase their tendency to seek feedback from supervisors. It proposes a useful managerial technique to promote subordinates’ feedback seeking behavior. Managers and human resources practitioners may want to encourage leaders to delegate more tasks and authority to subordinates in order to encourage them to seek feedback.

Our study also suggests that the effectiveness of the managerial technique of delegation depends on the power distance between leaders and subordinates. Delegating power to employees who believe there is a high power distance between them and the leaders makes them more likely to seek feedback from supervisors and so we particularly recommend that leaders in high power distance organizations should delegate more and increase subordinates’ involvement in decision-making process. Promoting the seeking of feedback by employees in this way is useful, because feedback helps employees to evaluate the adequacy and appropriateness of their work behavior and improve their performance (Ashford et al., 2003; Whitaker et al., 2007).

### Limitations and Future Research

There are some limitations to our study. First of all, the design we employed can be used to infer causal relationships. Further quasi-experimental or longitudinal research would be needed to determine causality. Second, we only investigated one method of promoting the seeking of feedback, namely use of delegation. In future researchers could explore other techniques for encouraging employees to seek feedback, for example mentoring (e.g., Allen et al., 2010). Third, our sample consisted of Chinese employees and power distance is considered to be high in China, so the generalisability of the findings is limited. We suggest that future researchers attempt to replicate our findings in samples from other cultures, particularly cultures with lower power distance.

## Conclusion

Leaders in organizations often face the challenge of encouraging subordinates to seek feedback, because feedback plays a critical role in improving job performance. Our study suggests that delegation is an effective way of encouraging subordinates to seek feedback, because it is psychologically empowering. Our results also highlight that the efficacy of delegation as a method of increasing the seeking of feedback is contingent on employees’ individual cultural value of power distance.

## Ethics Statement

All procedures performed in studies involving human participants were in accordance with the ethical standards of the institutional and/or national research committee and with the 1964 Helsinki declaration and its later amendments or comparable ethical standards with written informed consent from all subjects. This study was approved by the Human Protection and Research Ethics Committee at Business School, Beijing Normal University.

## Author Contributions

XZ and JQ substantially contributed to the conception and the design of the work as well as in the analysis and interpretation of the data. XZ prepared the draft, JQ, BW, and ZJ reviewed it critically and gave important intellectual input. Other contributing authors (JW and YW) reviewed the manuscript and gave comments on it.

## Conflict of Interest Statement

The authors declare that the research was conducted in the absence of any commercial or financial relationships that could be construed as a potential conflict of interest.
